# Actuation Behavior of Multilayer Graphene Nanosheets/Polydimethylsiloxane Composite Films

**DOI:** 10.3390/polym10111243

**Published:** 2018-11-09

**Authors:** Chunmei Zhang, Tianliang Zhai, Chao Zhan, Qiuping Fu, Chao Ma

**Affiliations:** 1 College of Chemistry and Materials Engineering, Guiyang University, Guiyang 550005, China; zhangzhang_87@126.com (C.Z.); taochao5@163.com (Q.F.); chaomagyu@126.com (C.M.); 2 Guizhou Building Material Quality Supervision Testing Center, Guiyang 550000, China; zhanchao0736@163.com

**Keywords:** graphene nanosheets, polydimethylsiloxane, dielectric constant, electromechanical actuation

## Abstract

The graphene nanosheets (GNS)/polydimethylsiloxane (PDMS) composite films with out-of-plane dielectric actuation behavior were prepared through a layer-by-layer spin coating process. The GNS-PDMS/PDMS composite films with 1~3 layers of GNS-PDMS films were spin coated on top of the PDMS film. The dielectric, mechanical, and electromechanical actuation properties of the composite films were investigated. The dielectric constant of the GNS-PDMS^3^/PDMS composite film at 1 kHz is 5.52, which is 1.7 times that of the GNS-PDMS^1^/PDMS composite film. The actuated displacement of the GNS-PDMS/PDMS composite films is greatly enhanced by increasing the number of GNS-PDMS layers. This study provides a novel alternative approach for fabricating high-performance actuators with out-of-plane actuation behavior.

## 1. Introduction

Recently, much attention has been paid to dielectric elastomers because they are lightweight, have mechanical resiliency, low cost, facile processability, direct voltage control, noiseless performance, scalability and shock tolerance [1,2,3]. Dielectric elastomers can directly convert electrical energy into mechanical work or vice versa, which made them promising materials for advanced electromechanical applications in actuators, generators and sensors [4,5,6]. Generally, dielectric elastomer actuators consist of an elastomer film sandwiched between two thin and compliant electrodes, thereby forming a capacitor capable of energy transduction. When an external voltage is applied to the electrodes, electrostatic forces are created. Electrostatic pressure acting on the film squeezes the elastomer in thickness, and as the elastomer is incompressible it consequently expands in the in-plane direction. The driving force for the actuation process is the resulting reduction in charge density when the electrode area is enlarged. When the external voltage is switched off, the elastomer film returns to its original shape. 

For dielectric elastomer actuators, the elastomer matrix is very important because it governs dielectric constant, dielectric breakdown strength and obtainable strain. Silicone elastomers, including the polydimethylsiloxane (PDMS), are one of the most frequently used materials for dielectric elastomer actuators due to their good elasticity, reliability and fast response speed [7,8,9,10,11]. They produce repeatable, reproducible actuation upon activation, they show little tendency for Mullins (stress softening) and ageing effects and they have been shown to actuate (at low strains) to more than 4 million cycles, in some instances more than 400 million cycles, without failure [12,13]. They also possess significantly lower viscous losses than acrylics, indicating that they can be operated at higher frequencies with lower losses and less heat generation. Furthermore, silicone elastomers can be operated at a broader temperature range and possess inherent softness and stability. However, the silicone elastomers are non-polar, which demonstrate limited deformation under electrical field due to their inherent low dielectric constant [14]. This means that silicone elastomers, despite possessing superior mechanical properties compared to other dielectric elastomers, do not reach their full potential when used as actuators.

To enhance the actuation efficiency of the silicone elastomer actuators, the dielectric constant of the system needs to be increased. The addition of a filler with high constant to the elastomer system is one of the most investigated solutions to this challenge. Inorganic particles (TiO_2_ and titanates) [7,15], metals (iron, cobalt and titanium complexes) [16], organo-clays (montmorillonite) [17], as well as conductive fillers (carbon black, carbon nanotubes, and exfoliated graphite) [18,19] have been added in silicone matrix to investigate the actuator application. For the optimal system, a balance will have to be found between reinforcement and constant increase while not compromising the elastic modulus. Compared with the traditional method of adding a mass of high-dielectric-constant ceramics, the way of loading a much reduced quantity of conductive fillers can effectively maintain the mechanical flexibility of silicone elastomer matrices [19]. Meanwhile, this way is beneficial for improving the systematic dielectric constant of composites according to the electrical percolation and microcapacitor theory [20,21].

Since the surprising milestone of the 2010 Nobel Physics Prize awarded for the preparation of graphene, the two-dimensional material has been widely used as a functional filler in polymer nanocomposites for its extraordinary mechanical, thermal and electrical properties [22]. Graphene can cause a crucial improvement in the electrical, mechanical, and thermal properties of the resulting graphene/polymer composites at very low loadings. Graphene-based electroactive polymers are being developed for promising applications in electromechanical systems. Kim et al. [23] proposed an actuator consisting of a dielectric elastomer substrate and multiple stacked graphene electrodes. The actuator had a stretch ability of 25% thanks to the mechanical properties of the graphene electrode. Tungkavet et al. [24] studied the electromechanical properties of graphene/gelatin hydrogel composites for use in actuator applications. They found that the graphene/gelatin composite with only 0.1 vol % filler loading delivered the greatest deflection distance and dielectrophoresis force. Chen et al. [25] demonstrated that polyurethane dielectric elastomer filled with titanium dioxide functionalized graphene displayed evident electric stimulus response and electric field induced strain.

In this paper, we developed graphene nanosheets (GNS)/polydimethylsiloxane (PDMS) composite films through a layer-by-layer spin coating process for electroactive actuators. The effect of the number of GNS-PDMS layers on the mechanical, dielectric, and electromechanical actuation performance of the resulting multilayer GNS-PDMS/PDMS composite films was investigated. The addition of GNS to PDMS effectively improves the dielectric constant of the composite film, and the actuated displacement of the composite films is greatly enhanced by increasing the number of GNS-PDMS layers.

## 2. Materials and Methods 

### 2.1. Preparation of Reduced Graphene Oxide/DMF Solution

The preparation of graphene oxide (GO) was described in our previous work [26]. The obtained GO (30 mg) was dispersed into dimethylformamide (DMF) (100 mL) by a probe ultrasonicator for 1 h in an ice-water bath to get a uniform GO suspension (3 mg/mL). Then, hydrazine hydrate was added (weight ratio hydrazine hydrate/GO = 7/10). The reduction reaction was conducted at 90 °C for 24 h under magnetic stirring. After cooling to room temperature, the graphene nanosheets (GNS)/DMF solution was obtained.

### 2.2. Preparation of GNS/THF Solution

The GNS/DMF suspension was vacuum filtrated through a funnel. The filtered GNS was washed with tetrahydrofuran (THF) five times to remove any residual DMF. Then the wet GNS was transferred into THF to get GNS/THF suspension with a concentration of 5 mg/mL.

### 2.3. Preparation of GNS/PDMS/THF Solution

The polydimethylsiloxane (PDMS) prepolymer (10 g) (184 Silicone Elastomer, Dow Corning, Midland, MI, USA) was mixed with THF (5 mL) to obtain a mixture of PDMS/THF. The resulting PDMS/THF mixture was mixed with the GNS/THF solution (6 mL) using a vortex mixer for 3 min and followed by ultrasonication for 1 h. The curing agent (1 g) (Dow Corning, Midland, MI, USA) for PDMS was then added into the mixture. After 1 min of mechanical stirring with vortex mixer, and 10 min bath sonication treatment, the GNS/PDMS/THF solution was obtained.

### 2.4. Preparation of Multilayer GNS-PDMS Composite Films

The PDMS prepolymer and curing agent (weight ratio 10:1) were mixed in a 50 mL centrifuge tube according to the manufacturer’s instructions. The centrifuge tube was placed in a vacuum oven and degassed for 5~10 min at room temperature. Then, the PDMS was spin coated on a silicon wafer at 3000 rpm for 30 s, and the silicon wafer was transferred to a hot oven immediately at 150 °C for 30 min to fully cure the PDMS. After the silicon wafer cooled down to room temperature, the GNS/PDMS/THF solution was spin coated on top of the PDMS layer with the same procedure described above and cured to get the double-layer GNS-PDMS/PDMS composite film, which is denoted as GNS-PDMS^1^/PDMS. The sample with two layers of GNS-PDMS films coated on top of the PDMS layer is denoted as GNS-PDMS^2^/PDMS. The sample with three layers of GNS-PDMS films coated on top of PDMS layer is denoted as GNS-PDMS^3^/PDMS. For comparison, two layers of PDMS film were also prepared by the same procedure and is denoted as double-layer PDMS film.

### 2.5. Characterization

For all the tests described below, at least three replicates were measured for each sample, and the average results were reported [27]. The dielectric constant and dielectric loss (tan δ) of the films were measured using an E4980A LCR meter (Agilent Technologies, Inc., Santa Clara, CA, USA) at room temperature with a 16451B dielectric test fixture (Agilent Technologies, Inc., Santa Clara, CA, USA). Mechanical properties were characterized by an electronic tensile machine (JPL-2500, Jiangdu Jingcheng Testing Instrument, Yangzhou, China) at room temperature according to the ASTM D882 standard for tensile testing of thin plastic films. The breakdown field strength of the films was tested by an AMS-10B2 high voltage amplifier (Matsusada Precision Inc., Shiga, Japan). 

**Figure 1:**
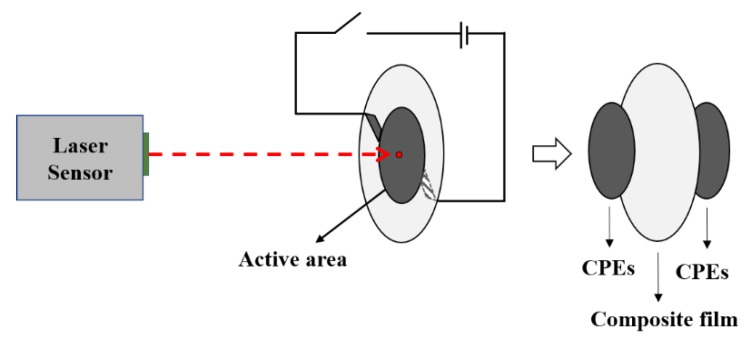
Schematic of the electromechanical response testing set-up for the composite films.

The out-of-plane actuation properties of the films were characterized under applied electric field. The testing set-up was schematically illustrated in Figure 1. The electric field was applied by the high voltage amplifier. The voltage was turned on for 30 s and off for 30 s and repeated many times with gradual increase of the voltage until the film was broken or the displacement reached the extreme of the laser triangulation sensor. Both sides of the composite films were pasted with carbon paste electrodes (CPEs, EXLUB G310, Dongguan Excellent Lubrication Technology, Dongguan, China) in a circular shape to generate a uniform electric field throughout the active area. The displacement of the composite films was measured using a high-speed laser triangulation sensor (MICROTRACK LTC-025-02, MTI Instruments, Albany, NY, USA). The diameter of the circular active area was 16 mm. The films were biaxially stretched with 5% strain prior to electromechanical characterization to get a stable electromechanical response [28,29].

## 3. Results and Discussion

**Figure 2:**
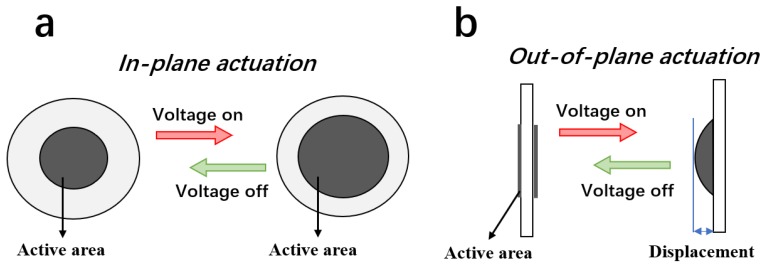
Schematic of the (**a**) in-plane actuation and (**b**) out-of-plane actuation.

The in-plane actuation is the most typical electromechanical response for dielectric elastomers. Generally, when an electric field is applied, the dielectric elastomer film contracts along the thickness direction due to the electrostatic charges, and extends along the transverse direction, as shown in Figure 2a. The compression force denoted as *σ_z_* is the Maxwell stress [30], which is calculated by Equation (1),

(1) $$\sigma_{z}=\varepsilon_{0}\varepsilon_{r}{\left(\frac{V}{t}\right)}^{2},$$

where *ε*_0_ is the dielectric constant of the air and *ε_r_* the relative dielectric constant of the elastomer. *V* represents the applied voltage and *t* denotes the thickness of the elastomer. 

The field-induced strain *S* in the transverse direction is calculated by Equation (2),

(2) $$S=\frac{\sigma_{z}}{Y}\times 100\%,$$

where *Y* represents the Young’s modulus of the elastomer.

From Equations (1) and (2) we can find that the elastomer with higher *ε_r_* and lower *Y* can generate larger strain with better actuation ability. Usually, a large pre-strain is needed to obtain the in-plane actuation [8]. The out-of-plane actuation is the electromechanical response of the elastomer that deforms along the thickness direction, as shown in Figure 2b. When the dielectric elastomer is fixed by a frame with low or without pre-strain, the out-of-plane actuation could be observed [23]. When an elastomer film was prepared with different actuation abilities on its two sides, the film would upheave to the side with greater actuation ability. The out-of-plane actuated displacement could be improved by enhancing the dielectric constant and reducing the Young’s modulus of the film.

The thickness, dielectric constant, dielectric loss (tan δ), and breakdown field strength of the GNS-PDMS/PDMS composite films are given in Table 1. The thickness of the double-layer PDMS film is 78.2 μm, which is thicker than that of the GNS-PDMS^1^/PDMS composite film with a thickness of 71.4 μm. This is because the viscosity of the PDMS prepolymer is higher than that of the GNS/PDMS/THF solution. During the spin coating procedure, higher viscosity leads to a thicker film. The dielectric constant of GNS-PDMS^1^/PDMS composite film at 1 kHz is 3.34, which is much higher than that of the double-layer PDMS film with only 2.22, indicating the addition of GNS greatly improves the dielectric constant of the composite film. The thickness and dielectric constant of the composite films increase with the number of layers. The dielectric constant of the GNS-PDMS^3^/PDMS composite film at 1 kHz is 5.52, which is 1.7 times that of the GNS-PDMS^1^/PDMS composite film. This indicates the dielectric constant of the composite films is enhanced by the GNS and strongly dependent on the number of layers. Besides, the dielectric loss of the GNS-PDMS/PDMS films at 1 kHz decreases with the number of GNS-PDMS layers. The breakdown field strength of the double-layer PDMS film is higher than 164 MV/m, because the real value is beyond the measuring range. The breakdown field strength of the GNS-PDMS/PDMS composite films significantly decreases with an increase in the number of GNS-PDMS layers, which decreases from 109 to 64 MV/m when the number of GNS-PDMS layers increases from one to three.

**Figure 3:**
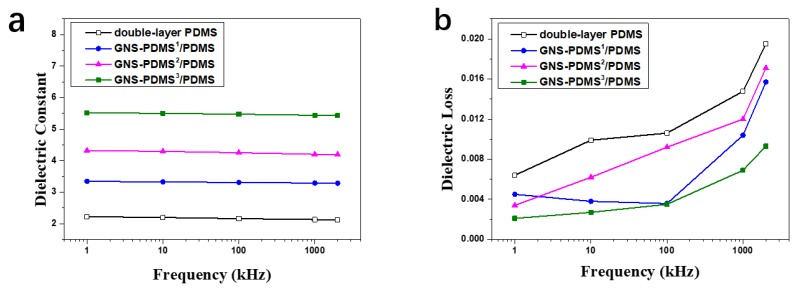
Dielectric constant (**a**) and dielectric loss (**b**) of the films as a function of frequency.

The dielectric constant and dielectric loss of the films as a function of frequency are shown in Figure 3. It can be found that the dielectric constant of each sample slightly decreases with the increase of frequency. For instance, the dielectric constant of the GNS-PDMS^3^/PDMS composite film is 5.52 at 1 kHz, and it slightly decreases to 5.44 at 1000 kHz. The dielectric loss of each sample increases with the increasing frequency.

**Figure 4:**
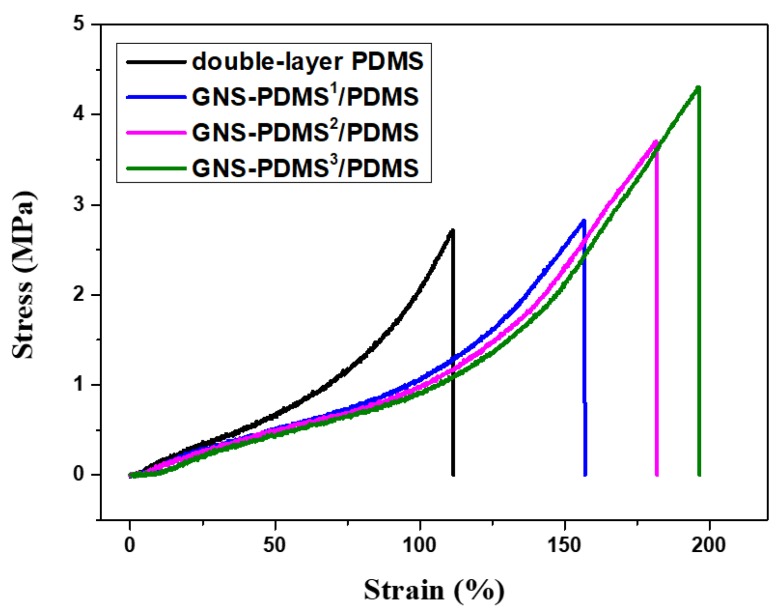
Tensile stress-strain curves of the films.

The tensile stress-strain curves of the films are presented in Figure 4, and the mechanical properties are given in Table 2. It is obvious that the addition of GNS to PDMS leads to a softening effect. The GNS-PDMS^1^/PDMS composite film showed lower Young’s modulus and higher elongation at break than that of the double-layer PDMS film. The softening effect of the GNS to the PDMS matrix can be attributed to the residual hydroxyl groups on GNS that decrease the crosslinking density of the PDMS matrix. The crosslinking reaction of the PDMS is based on the hydrosilation, which occurs between the active hydrogens on the crosslinking agent and the vinyl groups on the linear PDMS prepolymer in the presence of a catalyst [31]. The GNS were obtained by the chemical reduction, which resulted with a certain amount of residual hydroxyl groups on the surface [32]. The hydroxyl groups reacted with the vinyl groups on the linear PDMS prepolymer. As a result, the crosslinking density of the PDMS matrix was reduced. Therefore, the more GNS-PDMS layers are coated on top of the PDMS layer, the greater softening effect is observed. The composite films exhibit reduced Young’s modulus and enhanced elongation at break with an increasing number of GNS-PDMS layers. For the composite film with three layers of GNS-PDMS, i.e., GNS-PDMS^3^/PDMS, the Young’s modulus decreases by 23.2% and the elongation at break increases by 25.2% in comparison to that of the GNS-PDMS^1^/PDMS film. This softening effect is beneficial for improving the out-of-plane actuation property because larger strain can be generated for a film with lower Young’s modulus according to Equation (2). In addition, the GNS also shows a reinforcing effect on the composite film. The tensile strength at break of the GNS-PDMS^1^/PDMS composite film is 2.82 MPa, it increases to 3.71 MPa for the GNS-PDMS^2^/PDMS composite film, and further to 4.30 MPa for the GNS-PDMS^3^/PDMS^1^ composite film.

**Figure 5:**
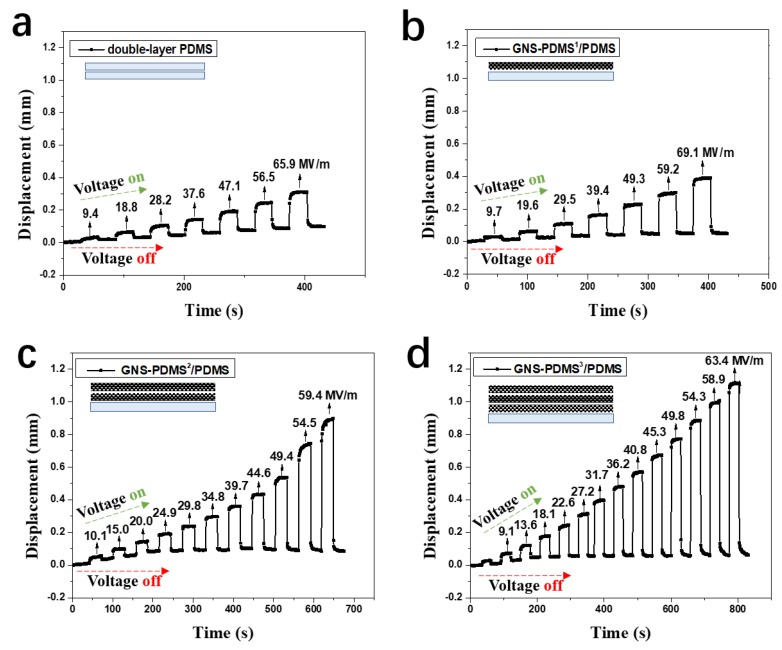
Actuated displacement as a function of time and electric field intensity: (**a**) Double-layer polydimethylsiloxane (PDMS); (**b**) GNS-PDMS^1^/PDMS; (**c**) GNS-PDMS^2^/ PDMS; and (**d**) GNS-PDMS^3^/PDMS.

Figure 5 shows the out-of-plane actuation displacement of the samples as a function of time and electric field intensity. The displacement was obtained by measuring the distance that the center point of the active area deviated from its original spot, as shown in Figure 2b. The electric field intensity was calculated by dividing voltage by film thickness. The displacement of the films increases with an increase in electric field intensity, as shown in Figure 5. The actuated displacement of the GNS-PDMS^1^/PDMS composite film is 0.16 mm at an electric field of 39.4 MV/m, and it increases to 0.39 mm at 69.1 MV/m, which is higher than that of the double-layer PDMS film exhibiting a 0.31 mm displacement at 65.9 MV/m. The displacement of the composite films is greatly improved by increasing the number of GNS-PDMS layers. For example, the displacement of GNS-PDMS^3^/PDMS composite film at 63.4 MV/m is 1.12 mm, which is 2.9 times that of the GNS-PDMS^1^/PDMS composite film at an even higher electric field of 69.1 MV/m. 

**Figure 6:**
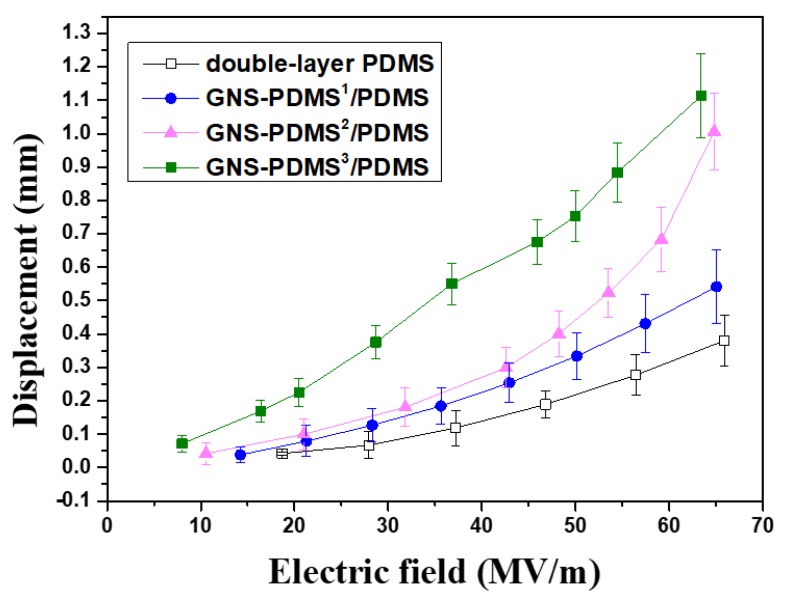
Displacement of the films as a function of electric field intensity

The displacement of the films as a function of electric field intensity is also shown in Figure 6 for convenient observation and comparison. The displacement increases with the increasing electric field intensity, as well as the number of GNS-PDMS layers. The GNS-PDMS^3^/PDMS composite film with three layers of GNS-PDMS films exhibits the highest displacement. According to the dielectric constant and the Young’s modulus results, the GNS-PDMS^3^/PDMS composite film possesses the highest dielectric constant and lowest Young’s modulus, which results in the highest displacement with the best actuation ability. The results indicate that the addition of GNS improves the actuation property of the composite film, and the out-of-plane actuation displacement is greatly enhanced by increasing the number of GNS-PDMS layers.

## 4. Conclusions

The GNS/PDMS composite films with out-of-plane actuation behavior were prepared by a layer-by-layer spin coating process. The GNS-PDMS/PDMS composite film shows higher dielectric constant than that of the double-layer PDMS film, indicating the addition of GNS greatly improves the dielectric constant of the composite film, which is beneficial for improving the actuation property. The dielectric constant of the GNS-PDMS/PDMS composite films increases dramatically with the number of GNS-PDMS layers. The composite films show reduced Young’s modulus and increased elongation at break with an increased number of GNS-PDMS layers. The increased dielectric constant and reduced Young’s modulus are beneficial to improve the actuation ability. Thus, the GNS-PDMS^3^/PDMS composite film with the highest dielectric constant and lowest Young’s modulus exhibits the highest displacement. This study offers a novel method to fabricate actuators with out-of-plane actuation behavior.

## Figures and Tables

**Table 1:** Thickness, dielectric constant, dielectric loss, and breakdown field strength of the films.

Sample	Thickness (μm)	Dielectric Constant (at 1 kHz)	Dielectric Loss (at 1 kHz)	Breakdown Field Strength (MV/m)
Double-layer PDMS	78.2 ± 1.5	2.22	0.0064	>164
GNS-PDMS^1^/PDMS	71.4 ± 1.2	3.34	0.0045	118 ± 2
GNS-PDMS^2^/PDMS	96.4 ± 1.3	4.32	0.0034	87 ± 1
GNS-PDMS^3^/PDMS	111.6 ± 1.0	5.52	0.0021	64 ± 1

**Table 2:** Mechanical properties of the films

Sample	Tensile Strength at 100% Strain (MPa)	Tensile Strength at Break (MPa)	Young’s Modulus (kPa)	Elongation at Break (%)
Double-layer PDMS	2.07	2.72	14.6	111.3
GNS-PDMS^1^/PDMS	1.07	2.82	11.2	156.8
GNS-PDMS^2^/PDMS	0.97	3.71	8.9	181.7
GNS-PDMS^3^/PDMS	0.91	4.30	8.6	196.3
